# Water as a Blood Model for Determination of CO_2_ Removal Performance of Membrane Oxygenators

**DOI:** 10.3390/membranes11050356

**Published:** 2021-05-12

**Authors:** Benjamin Lukitsch, Raffael Koller, Paul Ecker, Martin Elenkov, Christoph Janeczek, Markus Pekovits, Bahram Haddadi, Christian Jordan, Margit Gfoehler, Michael Harasek

**Affiliations:** 1 Institute of Chemical, Environmental and Bioscience Engineering, TU Wien, 1060 Vienna, Austria; raffael.koller@tuwien.ac.at (R.K.); paul.ecker@tuwien.ac.at (P.E.); markus.pekovits@tuwien.ac.at (M.P.); bahram.haddadi.sisakht@tuwien.ac.at (B.H.); christian.jordan@tuwien.ac.at (C.J.); michael.harasek@tuwien.ac.at (M.H.); 2 Institute of Engineering Design and Product Development, TU Wien, 1060 Vienna, Austria; martin.elenkov@tuwien.ac.at (M.E.); christoph.janeczek@tuwien.ac.at (C.J.); margit.gfoehler@tuwien.ac.at (M.G.)

**Keywords:** membrane oxygenation, carbon dioxide (CO_2_) removal, in vitro tests, in vivo tests, membrane modeling, computational fluid dynamic simulations, oxygenator development

## Abstract

CO_2_ removal via membrane oxygenators has become an important and reliable clinical technique. Nevertheless, oxygenators must be further optimized to increase CO_2_ removal performance and to reduce severe side effects. Here, in vitro tests with water can significantly reduce costs and effort during development. However, they must be able to reasonably represent the CO_2_ removal performance observed with blood. In this study, the deviation between the CO_2_ removal rate determined in vivo with porcine blood from that determined in vitro with water is quantified. The magnitude of this deviation (approx. 10%) is consistent with results reported in the literature. To better understand the remaining difference in CO_2_ removal rate and in order to assess the application limits of in vitro water tests, CFD simulations were conducted. They allow to quantify and investigate the influences of the differing fluid properties of blood and water on the CO_2_ removal rate. The CFD results indicate that the main CO_2_ transport resistance, the diffusional boundary layer, behaves generally differently in blood and water. Hence, studies of the CO_2_ boundary layer should be preferably conducted with blood. In contrast, water tests can be considered suitable for reliable determination of the total CO_2_ removal performance of oxygenators.

## 1. Introduction

Blood oxygenators, also known as artificial lungs, are needed to supplement respiratory function during cardiopulmonary bypass or to support patients with respiratory failure. A prerequisite for sufficient gas exchange is a large gas-exchange surface and efficient contact between the blood and membrane. To provide a large gas-exchange surface at the lowest possible priming volume, hollow fiber membrane packings are used [1]. In modern oxygenators, blood flows on the shell side of the packing while the fiber lumen are purged with O_2_. The transmembrane transfer of the respiratory gases (CO_2_ and O_2_) is hereby facilitated by the partial pressure difference between the blood and gas phase [2].

Although great efforts have been made to improve the biocompatibility of oxygenator circuits [3], serious side effects occur due to contact of blood with the artificial polymer surfaces. These side effects ultimately include reduced platelet function and survival as well as prolonged bleeding times after perfusion [4]. Consequently, the optimization of oxygenators aims for increasing gas exchange efficiency while reducing the membrane surface and blood priming volume [5]. 

For reviewing and studying optimized oxygenator designs, experimental methods are the most reliable. However, the use of blood significantly increases the effort and costs in many ways:

1. The use of blood is accompanied by animal suffering.
2. The use of blood is not permitted in all technical laboratories.
3. Test circuits get contaminated and must be rebuilt due to blood deposits.
4. Additional logistical challenges due to limited durability of blood.
5. Ethics committee approval must be obtained.

Water is a cheap, save and easy-to-handle substitute. However, water tests must be able to reasonably represent the CO_2_ removal performance observed with blood. Here, the difference in physical and chemical properties between blood and waster must be considered. While O_2_ solubility of blood differs significantly from that of water due to the binding of O_2_ to hemoglobin [6], the CO_2_ solubility in water and blood is subject to similar mechanisms. CO_2_ first dissolves physically and then reacts to carbonic acid (H_2_CO_3_), which dissociates to bicarbonate (HCO_3_^−^) [7]:

(1) $${\mathrm{CO}}_{2}+{\;H}_{2}O\;⇌{\;H}_{2}{\mathrm{CO}}_{3}⇌H^{+}+{\mathrm{HCO}}_{3}^{-}\;$$

**Figure 1:**
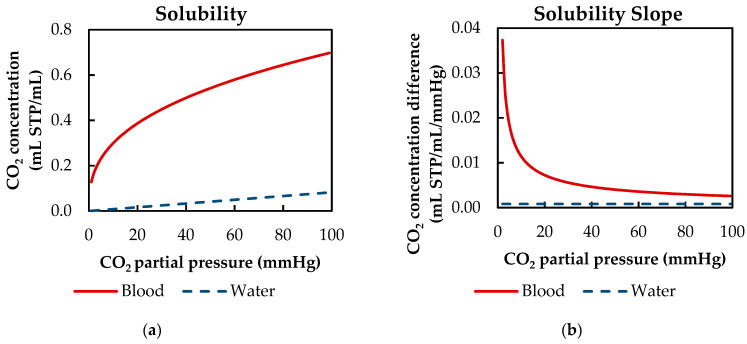
CO_2_ solubility behavior of water (Henry [9]) and blood (Loeppky et al. [10]): (**a**) CO_2_ concentration in dependency of CO_2_ partial pressure; (**b**) slope of CO_2_ concentration in dependency of CO_2_ partial pressure.

In order to shift the reaction equilibrium toward the production of bicarbonate, blood buffers the pH decrease, which is caused by the dissociation reaction. Hence, the total CO_2_ capacity of blood exceeds that of water. Examining the solubility curves of blood and water at clinically relevant CO_2_ partial pressures (40 to 100 mmHg) shows that the CO_2_ capacity of blood is up to 11 times higher (Figure 1a). Additionally, the reaction from CO_2_ to carbonic acid is accelerated by the catalytic enzyme carbonic anhydrase located in the red blood cells [7].

However, the effect of the buffer system and the enzymatic catalyst on the overall CO_2_ removal of oxygenators is limited because of two reasons. First, the slope of the solubility curve of blood and water at relevant venous levels is comparable. This is of importance, as the CO_2_ removal is primarily dependent on the concentration difference. At CO_2_ partial pressures from 40 to 100 mmHg, the slope of the solubility curve of blood is only four times higher than the slope of water (Figure 1b). Second, outside the red blood cells and in the absence of the catalyst carbonic anhydrase, the reaction from physically dissolved CO_2_ to carbonic acid and vice versa is slow, compared to the short residence time of blood in an oxygenator. Consequently, mostly physically dissolved CO_2_ is removed at the membrane [8].

Compared to water, the diffusion of CO_2_ components in blood is hindered by the presence of proteins [7]. This leads to an approximately 2.6 times higher diffusion rate for physically dissolved CO_2_ in water than in blood plasma [11].

**Figure 2:**
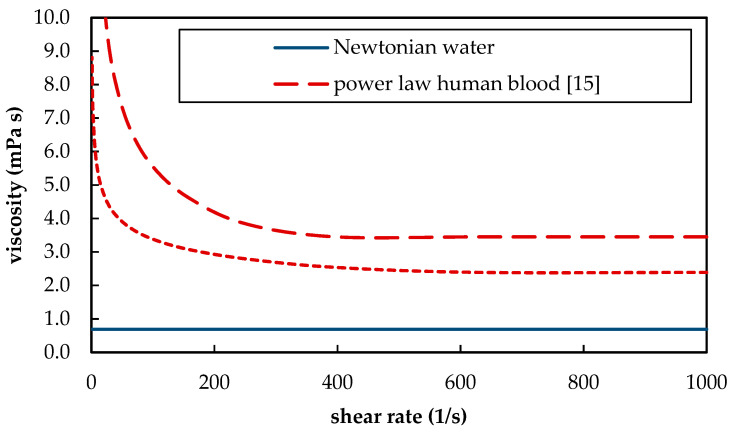
Rheologic behavior of blood and water at 37 °C. For representation of whole blood viscosity, the power law viscosity model was used for human [15] and porcine blood [16].

Furthermore, blood as a suspension of blood plasma and red blood cells shows shear thinning behavior. The shear thinning behavior hereby differs between different animal species [12] and depends, among other parameters, on the hematocrit [13]. At high shear rates, the whole blood viscosity converges toward its minimum (Figure 2). For human blood, this minimum is approximately 3.5 mPa s. In contrast, water is a Newtonian fluid and has a viscosity of 0.69 mPa s at 37 °C [14]. The different rheological behavior of blood and water (Figure 2) has two opposing influences on CO_2_ separation. On the one hand, lower viscosities result in more turbulent flow conditions. This could promote additional mixing. On the other hand, higher viscosities produce higher shear stress. Due to the higher shear stress, thinner boundary layers can be expected. Both additional mixing at lower viscosities (water) and thinner boundary layers at higher viscosities (blood) would result in an increase in CO_2_ removal.

To conclude, blood and water differ in CO_2_ solubility, CO_2_ diffusion rate and viscosity. These important parameters have partially opposing effects on the CO_2_ removal of oxygenators. This makes it difficult to evaluate the suitability and limitations of water as a blood substitute without using in-depth studies.

In recent research, multiple in vitro studies were conducted, using water as a blood substitute to determine the CO_2_ removal of an oxygenator. Hout et al. [17] investigated the dependency of CO_2_ removal on the sweep flow rate. This was done for two different oxygenator models. In order to be able to use water as a blood substitute, the gas exchange rate was normalized by the maximum CO_2_ removal rate of the respective oxygenator. By doing so, the measured CO_2_ removal rates can be made independent from the specific mass transfer characteristics of an oxygenator [18], which, in turn, are dependent on the used test fluid (blood/water). Hattler et al. [19] tested the CO_2_ and O_2_ transfer performance of a gas exchange catheter in vitro with water, and in vivo, using calves as a large animal model. However, the suitability of water is only discussed for the O_2_ exchange. Additionally, the in vitro and in vivo results cannot be compared directly due to the different hydrodynamic conditions. Consequently, a representative deviation of the CO_2_ removal rates determined with blood and water cannot be calculated. Svitek et al. [11] proposed a Sherwood model, allowing to predict the CO_2_ removal with blood, based on experiments with water, using an adapted diffusivity. To validate the model, in vitro tests with blood and water were conducted. Although the experimental results show comparable CO_2_ removal rates for blood and water they are not compared directly by the authors. The suitability of water for prediction of the CO_2_ removal of oxygenators is only discussed in the scope of the proposed conversion of water to blood data. Tabesh et al. [20] determined the CO_2_ removal rate of oxygenators via in vitro tests with porcine blood and water. To reduce the difference between the two CO_2_ removal rates of blood and water, N_2_ was blended into the saturation stream. This allowed to reduce the maximum deviation to 5%. Yet, the reason for the good fit between blood and water is not analyzed and discussed. Mihelc et al. [21] and Jeffries et al. [22] reported that the CO_2_ removal measured for an intracorporal membrane catheter correlates well (within 10% deviation) when comparing in vitro trials with water to in vivo trials with calves. The good agreement is attributed to the opposing effects on the CO_2_ removal rate induced by the different viscosities and CO_2_ solubilities of blood and water. An in-depth examination of this phenomenon is not conducted. Furthermore, they explicitly limit the suitability of water to their devices and consider the good correlation between water and blood to be fortuitous. A recommendation to use water for the in vitro determination of the CO_2_ removal rate is not given.

To summarize, water is commonly used as a blood substitute for the in vitro determination of the CO_2_ removal rate of oxygenators. Its suitability for determining the total CO_2_ removal rate has been confirmed by multiple independent research groups but has never been main focus of their published research. The CO_2_ solubility, CO_2_ diffusion rate and viscosity of water differ significantly from those of blood. Due to the complex interactions of these parameters, the reason for the suitability of water as a blood substitute for the determination of CO_2_ removal in oxygenators remains unclear. 

The scope of this research is to give a detailed comparison of CO_2_ removal rates gained from in vitro tests using water with data from in vivo tests using pigs as large animal models. To better understand the contribution of the differing CO_2_ solubility, CO_2_ diffusion and viscosity on the CO_2_ exchange, the CO_2_ concentration polarization in the boundary layer attached to the membrane is studied. It represents the main CO_2_ transport resistance and, therefore, characterizes the CO_2_-removal performance of oxygenators [2]. As the boundary layer cannot be resolved experimentally, computational fluid dynamic (CFD) simulations were conducted to investigate and compare the behavior of the boundary layer for blood and water. By comprising experimental and CFD results, this work aims to evaluate the suitability and limitations of water as a blood substitute for the determination of the CO_2_ removal rate of oxygenators.

## 2. Materials and Methods 

### 2.1. In Vivo and In Vitro Tests

The in vivo and in vitro results displayed in this study represent a secondary analysis of previously published data [8,16]. In vivo tests using pigs as large animal models were conducted to validate a CFD model that allows to predict the CO_2_ removal rate of oxygenators [16]. In vitro tests with water were conducted to evaluate the accuracy of CO_2_ removal prediction via blood gas analyzer measurements [8]. A comparison of the CO_2_ removal rate of water and blood, as presented in this study, was not conducted in the previous publications.

**Figure 3:**
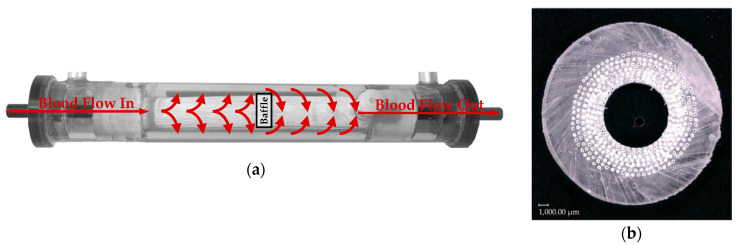
Prototype oxygenator: (**a**) Principle flow guidance; (**b**) slice of fiber potting to illustrate fiber arrangement—by Lukitsch et al. [16] (CC BY 4.0).

**Figure 4:**
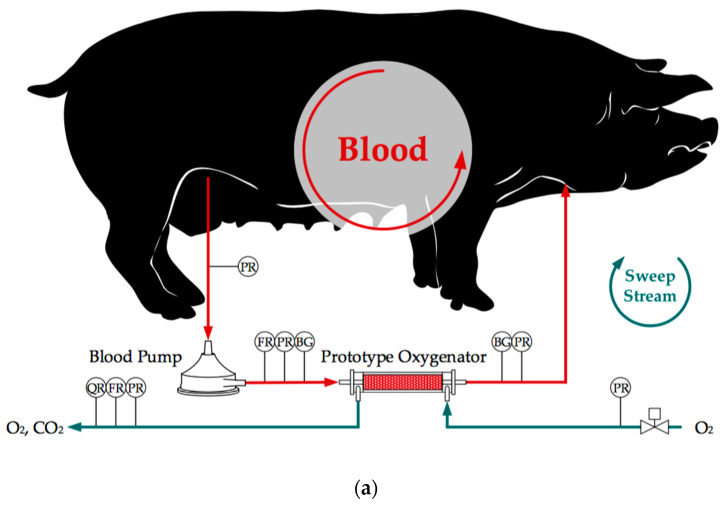

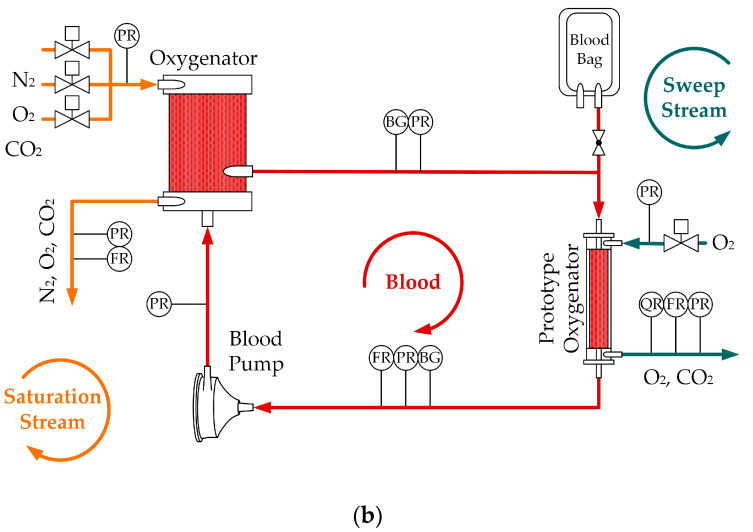
Schemes of the test setups showing the prototype oxygenator, blood pump, pressure sensors (PR), flow rate sensors (FR), sample ports for the blood gas analyzer (BG) and CO_2_ concentration sensor (QR): (**a**) scheme of in vivo loop—by Lukitsch et al. [16] (CC BY 4.0); (**b**) scheme of in vitro loop—by Lukitsch et al. [8] (CC BY 4.0).

In both test series, in vivo and in vitro, the CO_2_ removal rate of an oxygenator prototype was measured. The separation rate was determined at three blood/water flow rates (1000, 1300, and 1600 mL/min). This corresponds to the flow rate range of the oxygenator prototype, which was designed for the partial separation of metabolic CO_2_ production. For each of the three flow rates, three clinically relevant, pathologically elevated CO_2_ partial pressures levels (50, 70, and 100 mmHg) were investigated. This equals a total of nine measurement points. For each measurement point, three repetitions were performed. The CO_2_ removal rate was determined by measuring the flow rate (Defender 510, Bios DryCal, Mesa Laboratories, Inc., Lakewood, CO, USA) and CO_2_ concentration (BINOS 100 M, Emerson, St Louis, MO, USA) of the sweep gas flow exiting the prototype oxygenator. The examined prototype oxygenator (Figure 3) had a membrane surface of 0.06 m^2^ provided by commercial polymethylpentene (PMP) hollow fibers with an outer diameter and wall thickness of 380 and 90 µm, respectively. The schematic structure of the tests can be seen in Figure 4. Further experimental details can be found in our preceding publications (in vivo [16] and in vitro tests [8]).

### 2.2. Computational Fluid Dynamic Simulations

**Figure 5:**
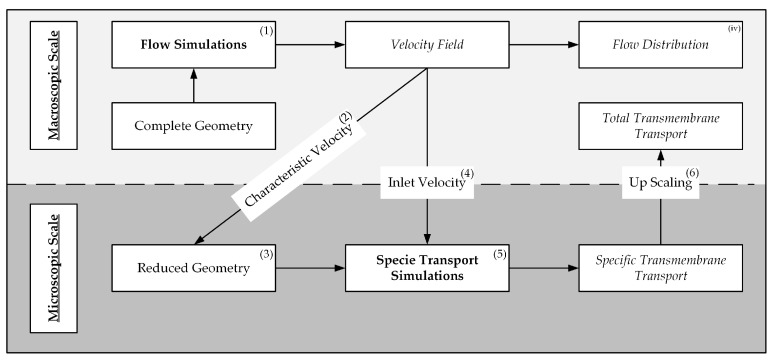
Workflow of the upscaling method for CFD based prediction of oxygenator CO_2_ removal performance—by Lukitsch et al. [16] (CC BY 4.0).

All CFD simulations were conducted with *OpenFOAM*^®^ *4.1* (ESI Group, Paris, France). The simulations were run on server nodes equipped with 32 core CPUs (16 cores in two physical modules, EPYC 7351, AMD, Santa Clara, CA, USA). For the CFD CO_2_ transport simulations, the geometry of the prototype oxygenator fiber packing was simplified to reduce the computational effort. Velocity boundary conditions of this reduced geometry were determined by means of an upscaling method [16] that uses CFD flow simulations of a complete or representative part of a hollow fiber module and samples the velocities within this packing. The velocity samples are, afterward, used to calculate an average velocity, which can be used to set the inlet velocity boundary condition of the reduced geometry. In doing so, the method allows to model the flow conditions in the reduced geometry to be representative for the flow regime within the complete fiber packing. CO_2_ transport simulations of the reduced geometry are then capable to give an accurate prediction of the average transmembrane flux. In Figure 5, the workflow of this upscaling method is illustrated. The upscaling method has been validated for a similar application. In the investigated case, the oxygenator CO_2_ removal rate predicted by CFD simulations of the reduced and complete geometry deviated by approx. 10% [16]. This deviation is comparable to the deviation between the experimentally determined CO_2_ removal rate and the CO_2_ removal rate determined by the CFD CO_2_ transport simulations of the reduced geometry (Section 3).

The CFD simulations were performed to extend the experimentally determined data. The experimental measurements of the nine measurement points were performed under steady-state conditions to allow for the three measurement repetitions. Therefore, only the steady-state flow and mass transport problem was considered in the CFD simulations. 

#### 2.2.1. Flow Simulation of the Complete Prototype Oxygenator (Macro Scale)

The velocity distribution of water in the complete prototype oxygenator was computed by solving the finite volume formulation of the steady incompressible Navier–Stokes equations, i.e., conservation of mass

(2) ∇(U)=0

and momentum. Here, **U**, *p*, *ρ* and µ denote the velocity field, pressure field, fluid density and dynamic viscosity.

(3) ∇(ρUU)−∇(µ∇U)=–∇p

**Figure 6:**
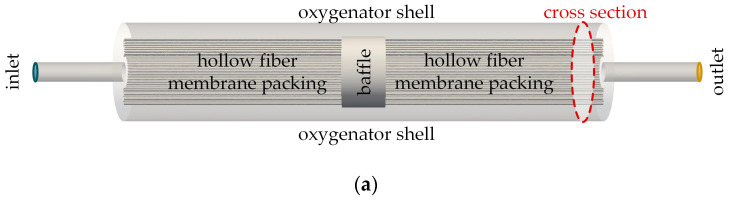

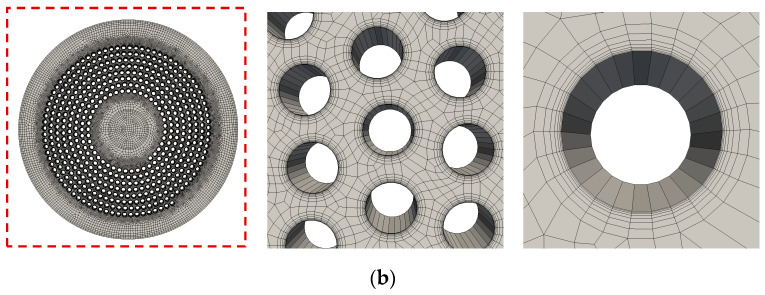
Geometry of CFD flow simulations: (**a**) Cross section of the prototype oxygenator, inside view; (**b**) cross section of the fiber packing with close up on the boundary layer mesh of the fibers.

The conservation of mass and momentum were solved with simpleFoam, the OpenFOAM implementation of the Semi-Implicit Method for Pressure Linked Equations (SIMPLE) algorithm. The computational mesh (Figure 6) was produced with Gambit 2.4.6 (ANSYS, Canonsburg, PA, USA), contained 32 Mio. hexahedron cells and was adapted directly from previous studies [16].

At maximum flow rates, the Reynolds number at the shell side inlet of the prototype oxygenator (Re_inlet_) is elevated, suggesting a transitional flow regime. Re_inlet_ was calculated using the diameter of the inlet pipe (4 mm) and average velocity in the inlet pipe at the maximum water flow rate (1600 mL/min).

(4) $$Re_{inlet}=\frac{u\times L}{\nu }\;\sim \;\frac{2.1\;m/s\times 4\;\mathrm{mm}}{1.0\times 10^{-6}\;m^{2}/s}\;\sim 8400$$

In contrast, the Reynolds number within the packing (Re_packing_) is low and indicates a laminar flow regime. Re_packing_ was calculated using the fiber spacing of the packing (200 µm) and average radial velocity within the packing at the maximum water flow rate (1600 mL/min). Average radial velocity was determined with the CFD flow simulations.

(5) $$Re_{packing}=\frac{u\times L}{\nu }\sim \frac{0.1\;m/s\times 200\;µm}{1.0\times 10^{-6}\;m^{2}/s}\sim 20$$

Due to the low Reynolds number in the region of interest (membrane packing), we expect limited influence of emerging turbulence on the CO_2_ removal performance. This assumption is supported by the experimental data. The laminar simulations allow for a reasonable prediction of the dependency of the CO_2_ removal rate on the blood/water flow rate (Section 3.1.2).

The transport equations (Equations (2) and (3)) were discretized using second order schemes (Van Leer [23]). At the beginning of the inlet pipe, uniform velocities were set corresponding to inlet flow rates of 1000, 1300 and 1600 mL/min. A no-slip velocity boundary condition was applied to all walls, including the membrane surfaces. A fixed uniform value of 0 Pa for the relative pressure was set at the end of the outlet pipe. All remaining boundary conditions for velocity and pressure were set to zero gradient (Neumann conditions). All boundary conditions of the flow simulations are summarized in Table 1. Kinematic viscosity of water was set to 6.96 × 10^−7^ m^2^/s (37 °C) [14].

The setup of the flow simulations computing the blood distribution in the complete oxygenator prototype is analogous to water flow simulations and described in detail in [8].

#### 2.2.2. CO_2_ Transport Simulations of the Simplified Packing (Micro Scale)

**Figure 7:**
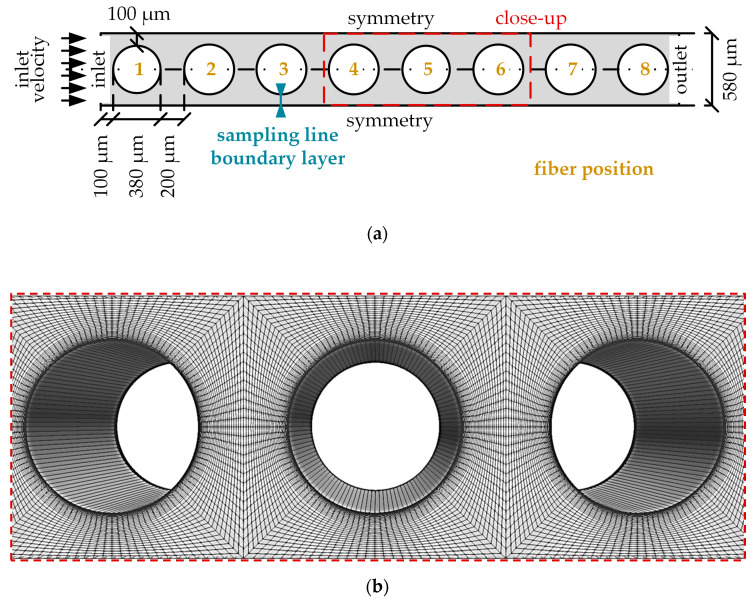
Simplified geometry of CFD CO_2_ transport simulations. (**a**) Fibers representing the eight fiber layers of the membrane packing positioned in a non-staggered arrangement, (**b**) close up of the computational mesh.

Details regarding the elaboration of the reduced geometry as well as the design and generation of the mesh can be taken from previous studies [8]. The reduced geometry consists of eight non-staggered fibers (Figure 7) representing the eight fiber mat layers built into the prototype oxygenator (Figure 3b). As the flow simulations show that the fibers are positioned mostly in cross flow mode, the inlet velocity was set to be perpendicular to the membrane packing (Figure 7a). The computational mesh, including only the shell side of the packing, counts 32000 hexahedron cells. To adequately resolve the boundary layer, 20 successively refining cell layers were applied to the membrane surface. In this refinement region, the cell thickness ratio between the most outer cell layer and the most inner cell layer (attached to the membrane) is 5 to 1 (Figure 7b). The cell thickness of the most inner cell equals 0.7 µm.

CO_2_ transport simulations have been performed using an inhouse solver, membraneFoam [24]. It is based on the open-source code OpenFOAM^®^ v4.1 (ESI Group, Paris, France) and implemented as a multi-region solver where the single regions are separated by a membrane. The solver balances the transport equations of velocity, pressure, density, energy and mass fraction for each single region separately. However, the regions are interlinked via the transmembrane transport, which is implemented as a volumetric source term in all of the transport equations. Transmembrane transport is calculated for all cells attached to the membrane. It is computed based on the membrane area of the cell (A) and the permeance (P). As the driving force, the partial pressure difference between the computational cell and an adjacent cell in the neighboring region at the other side of the membrane is utilized. If the membrane area of these two cells does not fully overlap (non-conformal mesh between the regions), partial pressure (p_CO_2__) can be interpolated from cells that are close by, adding further flexibility to the design of the mesh. A detailed description of the solver implementation as well as the mathematical formulation of the governing equations are provided by Haddadi et al. [24].

Previous findings indicated only a slight increase in CO_2_ partial pressure (p_CO_2__) on the sweep gas side [25]. Consequently, in this work, membraneFoam was used in a single region mode, setting the CO_2_ partial pressure on the sweep gas side constantly to 0 mmHg and reducing the computation of transmembrane CO_2_ transport (J_CO_2__) to the following:

(6) $$J_{{CO}_{2}}=P\times A\times \left(p_{{CO}_{2},Water}-0\right)$$

To solve the governing equations (transport equation of velocity, pressure, density, energy and mass fraction) the Pressure Implicit Method for Pressure-Linked Equations (PIMPLE) was used. They were discretized, applying second order schemes (linear, upwind). The inlet velocity was calculated using the upscaling method [8] and applied uniformly to the inlet patch. The inlet velocities for the reduced geometry corresponding to the blood and water flow rates off the complete geometry (prototype oxygenator) are summarized in Table 2.

The mass fractions of water and CO_2_ at the inlet were chosen to correspond to the CO_2_ partial pressures of 50, 70 and 100 mmHg, which were investigated in vitro and in vivo. On all walls and the outlet, Neumann conditions were used for the CO_2_ mass fraction. Symmetry conditions were applied to the sides of the geometry to account for the influence of adjacent fibers. The remaining velocity and pressure boundary conditions were set analogous to the flow simulation. All boundary conditions of the CO_2_ transport simulations are summarized in Table 3. 

CO_2_ was treated as a single species. The CO_2_ partial pressure was computed based on the mass fraction provided by the transport equation and by the use of the Henry’s model. The CO_2_ solubility (α_CO_2__) was set to 8.27 × 10^−4^ mL CO_2_ /mL/mmHg [9]. For the diffusion coefficient of CO_2_ (D_CO_2__) in water, a value of 2.38 × 10^−9^ m^2^/s was used [26]. Dynamic viscosity (µ) was set independently from CO_2_ concentration to 6.91 × 10^−4^ Pa s (at 37 °C) [14]. For the density (ρ) of water, a value of 993.33 kg/m^3^ (at 37 °C) was used [14]. 

The setup of the CO_2_ transport simulation of blood is analogous to the water CO_2_ transport simulation and described in detail in [8]. To enable a better overview, the material data used for blood and water are compared in Table 4.

**Figure 8:**
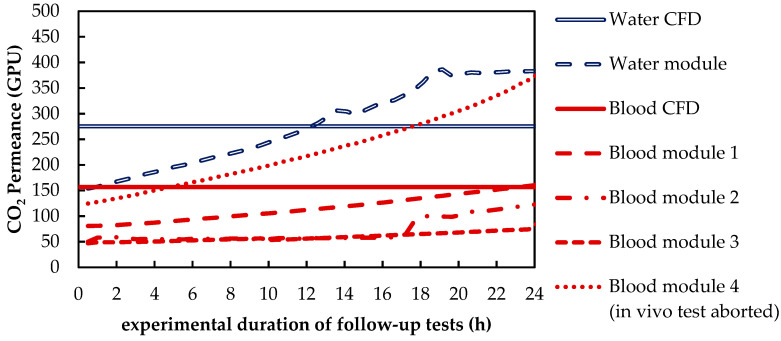
Increase in CO_2_ permeance during the follow up gas permeation measurements. Continuous lines: selected values for CFD CO_2_ transport simulations.

Before the trials, the permeances (P) of the prototype oxygenators were measured. The CO_2_ permeance (P_CO_2__) of unused fibers amounts to approx. 730 GPU. Follow-up tests showed that after in vitro or in vivo trials, the CO_2_ permeance of the prototype oxygenator is decreased considerably (50–150 GPU, 0 h of follow-up testing, Figure 8). During these follow-up tests, the permeances increased successively (to values of 90–380 GPU, 24 h of follow-up testing), most probably because of drying mechanisms induced by the sample gas flow (Figure 8). This observation is consistent with condensate water detected on the lumen side shortly after the beginning of the in vitro or in vivo trials. Both observations (successive increase in permeance and condensate on lumen side) additionally indicate wetting of the pores within the membrane. Furthermore, water residues also remain on the shell side of the membrane packing due to rinsing of the prototype oxygenator after the trials (porcine blood test) or due the trials themselves (water tests).

The recorded wetting of the lumen, the membrane pores and the shell reduce the permeances measured in the follow-up tests. However, the permeances set in the CO_2_ transport simulations must only account for the wetting of the lumen and pores, as the shell side (equivalent with blood/water side) CO_2_ transport resistance is resolved by the transport equations themselves. Thus, permeances required for CO_2_ transport simulations cannot be accurately measured for two reasons. First, the transport resistance of the wetted lumen and pores cannot be measured separately from the transport resistance present in the shell. Second, the drying of the shell results in the drying of the lumen and membrane pores to an uncertain extent, affecting the measured permeances.

Consequently, the permeances used in the simulations were chosen in order to fit the transmembrane CO_2_ flux of the simulation and experiments at a p_CO_2__ of 70 mmHg and a water/blood flow rate of 1300 mL/min. To keep the deviations between simulations and experiments below 10%, different permeances had to be chosen for blood and water. CO_2_ permeance with water (275 GPU) is 1.8 times higher than CO_2_ permeance with blood (157 GPU). This could be explained by the blood residues adding an additional transport resistance. The identified permeances lie within the measured range of pure gas permeances determined for the used membranes in the follow-up tests. A comparison of the permeances used in the CFD CO_2_ transport simulations and the permeances measured in the follow-up tests is given in Figure 8.

## 3. Results and Discussion

### 3.1. In Vivo and In Vitro Tests

In the following section, the CO_2_ removal rate of the prototype oxygenator determined in vivo with porcine blood and in vitro with water is compared. Additionally, the experimental results are used to validate the CFD simulations of CO_2_ transport in porcine blood and water.

#### 3.1.1. CO_2_ Removal Rate of In Vivo Porcine Blood and In Vitro Water Tests

**Figure 9:**
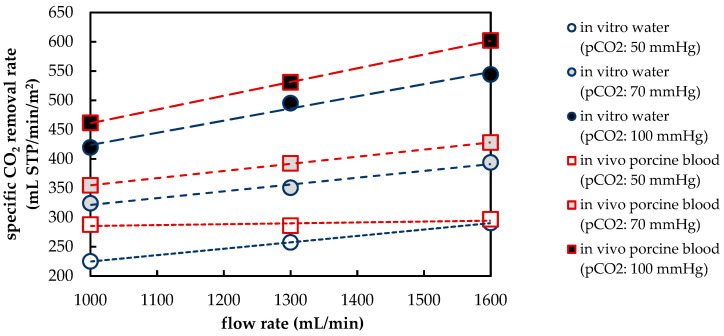
Comparison of experimentally determined CO_2_ removal. Red squares: in vivo studies with porcine blood; blue circles: in vitro studies with water.

Figure 9 summarizes the results of the in vivo tests with porcine blood and the in vitro tests with water. It shows the average CO_2_ removal of the prototype oxygenator for the nine measurement points (Section 2.1). The CO_2_ removal determined with porcine blood is generally higher than the CO_2_ removal of water. This is discussed in detail in Section 3.2.3. In both tests, CO_2_ removal increases with higher CO_2_ partial pressure (p_CO_2__) or higher blood/water flow rate. The dependency of CO_2_ removal on the flow rate increases with a higher p_CO_2__. This can be explained by an increased availability of physically dissolved CO_2_ at a higher p_CO_2__ and associated higher pH levels [7]. Since physically dissolved CO_2_ can be regarded as the main CO_2_ component removed via the membrane surface [8], higher availability of physically dissolved CO_2_ offers a larger potential of CO_2_ removal increase via flow rate increase.

The average deviation (ε) of the CO_2_ removal rate determined in vitro with water (J_CO_2___,water_) from that determined in vivo with blood (J_CO_2___,blood_) was calculated using the following equation:

(7) $$\varepsilon =\frac{\left(J_{{CO}_{2},Water}-J_{{CO}_{2},Blood}\right)\;}{J_{{CO}_{2},Blood}}\;$$

Average deviation is shown for the nine measurement points in Table 5. For p_CO_2__ ≥ 70 mmHg, ε is homogenously distributed and equals approximately 9%. At p_CO_2__ of 50 mmHg, ε shows a dependency on the flow rate. The deviation is highest at 1000 mL/min (21.8%) and decreases monotonously to a value of 2.1% at a flow rate of 1600 mL/min. 

However, the elevated ε at a p_CO_2__ of 50 mmHg and flow rate of 1000 mL/min (21.8%) could be caused by increased measurement errors, as at this measurement point, ε is two times the average and twice as large as the second largest deviation (Table 5). 

Furthermore, the dependency of ε on the flow rate is caused by the negligible, small dependency of the CO_2_ removal rate on the blood flow rate, measured in the in vivo trials at a p_CO_2__ of 50 mmHg. This small, recorded dependency can only be justified physically to a limited extent since at a p_CO_2__ of 50 mmHg, a dependency of the CO_2_ separation rate on the flow rate is predicted by both in vitro water tests and CFD CO_2_ transport simulations of blood (Section 3.1.2). Furthermore, Sherwood correlations also suggest a similar dependency [27]. In summary, this indicates increased measurement errors at a p_CO_2__ level of 50 mmHg. 

#### 3.1.2. Validation of CFD CO_2_ Transport Simulations for Porcine Blood and Water

**Figure 10:**
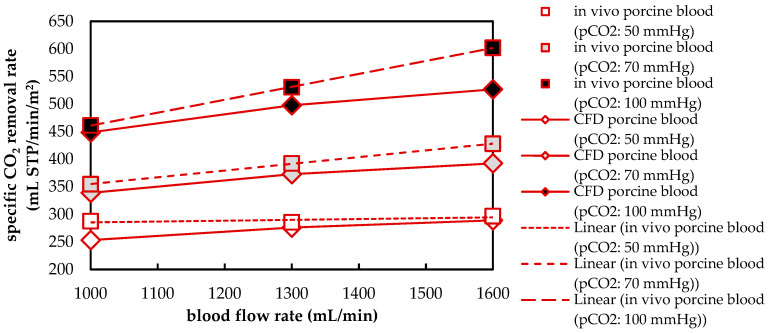
Comparison of CO_2_ removal determined with experiments and CFD simulations for blood.

To validate the CFD CO_2_ transport simulations, the numerically predicted CO_2_ removal rate is compared with the experimentally determined CO_2_ removal rate. Figure 10 shows a comparison of the experimental and computational results for porcine blood. In general, the results agree well. Average deviation of the numerically from the experimentally determined separation rate is 6%. However, a stronger deviation has to be noted at higher p_CO_2__ (100 mmHg) and higher flow rates (1600 mL/min). This can be attributed partly to an increased standard deviation (σ) of p_CO_2__ during the in vivo trials at a level of 100 mmHg (σ = 7.8 mmHg), compared to the other two p_CO_2__ levels (p_CO_2__: 50 mmHg, σ = 2.7 mmHg; p_CO_2__: 70 mmHg, σ = 3.8 mmHg). The dependence of the CO_2_ removal rate on the blood flow rate can be adequately described by the upscaling method used.

**Figure 11:**
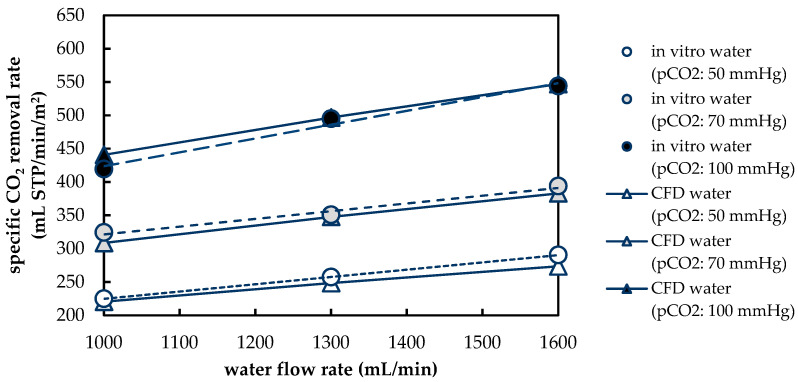
Comparison of CO_2_ removal determined with experiments and CFD simulations for water.

Figure 11 compares the experimentally and numerically determined CO_2_ removal rate for water. The results are in satisfactory agreement. The deviation of the experimentally and numerically determined CO_2_ removal rate is, on average, 3%. The dependence of CO_2_ removal on p_CO_2__ and water flow rate can be adequately described by the CFD model and the used upscaling method. 

The good agreement between the experimental and numerical results for both porcine blood and water suggests that the CFD models are suitable for detailed studies of the boundary layer. The latter determines the CO_2_ removal performance of an oxygenator since it can be considered the main transport resistance of the respiratory gas exchange.

### 3.2. Computational Fluid Dynamic—CO_2_ Transport Simulations

In the following section, the results of the CO_2_ transport simulations of porcine blood and water are summarized. Furthermore, the behavior of the boundary layer at different flow rates and CO_2_ partial pressures is discussed. Finally, the influence of the CO_2_ diffusion coefficient, CO_2_ permeance, CO_2_ solubility and viscosity on the deviation of the CO_2_ removal of porcine blood and water is evaluated.

#### 3.2.1. Flow and CO_2_ Partial Pressure Distribution in the Simplified Packing

**Figure 12:**
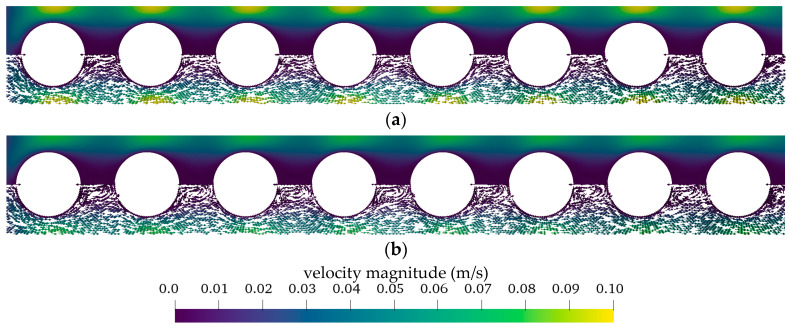
Flow distribution within the simplified packing (**a**) porcine blood (**b**) water. Bottom half of contour plot shows direction of velocity vectors.

Figure 12 compares the velocity distribution in the simplified fiber packing for porcine blood and water. Flow distribution is presented for an inlet velocity of 0.02 m/s (approx. 1300 mL/min). Porcine blood flow shows stronger velocity gradients and higher maximum flow rates (0.10 m/s) than water (0.08 m/s). Additionally, the velocity field indicates laminar flow profiles free of wakes. In comparison, the water flow distribution shows small wakes downstream of the fibers. However, in these areas, only low velocities (approx. 0.01 m/s) occur. In general, the flow behaves similarly at all fibers.

**Figure 13:**
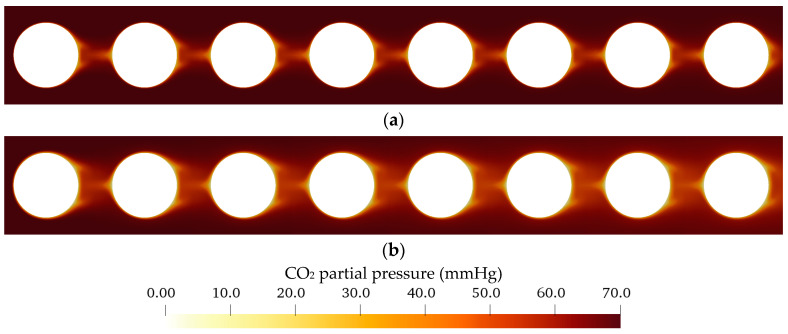
Distribution of CO_2_ partial pressure within the simplified packing (**a**) porcine blood (**b**) water.

In Figure 13, the CO_2_ partial pressure (p_CO_2__) contour plot of blood and water are compared for a uniform inlet p_CO_2__ of 70 mmHg. The CO_2_ partial pressure distribution in water shows a larger area with decreased p_CO_2__ than blood. This is most pronounced downstream of the fibers where the small wakes are positioned. This indicates that these wakes produce additional mixing. Furthermore, the higher CO_2_ diffusion rates of water could contribute to CO_2_ depletion in wider areas of the flow.

#### 3.2.2. Boundary Layer Study

**Figure 14:**
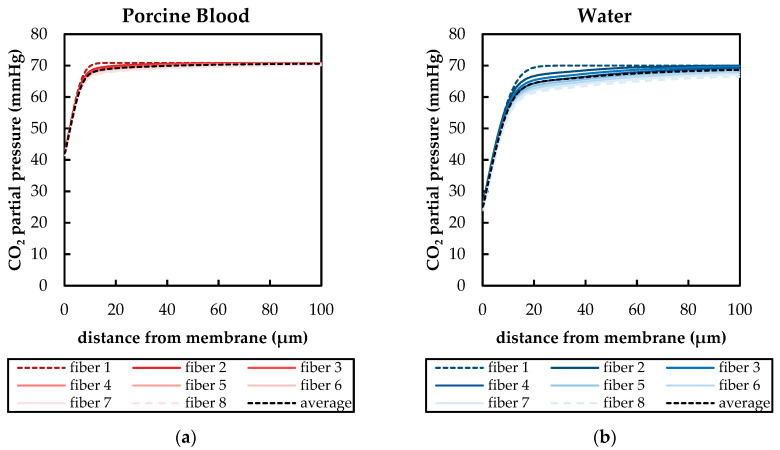
CFD boundary profiles of CO_2_ partial pressure at different fiber positions (sampling line illustrated in Figure 7a) for an inlet p_CO_2__ of 70 mmHg and inlet velocity of 0.02 m/s (approx. 1300 mL/min), fiber 1—first fiber at inlet, fiber 8—last fiber at outlet: (**a**) porcine blood, (**b**) water.

As can be seen in Figure 12 and Figure 13, flow and p_CO_2__ distribution depend only slightly on fiber position. They deviate strongest for the first fiber, as it is the only fiber which is not positioned in the slip-stream of another fiber. This can be also seen in Figure 14. It shows the p_CO_2__ profiles perpendicular to the main flow direction and the membrane wall for all eight fibers. The presented data were computed for a p_CO_2__ of 70 mmHg and an inlet velocity of 0.02 m/s (approx. 1300 mL/min). The sample lines of these profiles and the fiber positions are illustrated in Figure 7a. 

The p_CO_2__ profiles of blood (Figure 14a) depend less on fiber position than the p_CO_2__ profiles of water (Figure 14b). This could be attributed to the higher Re of the water flow, which promotes additional mixing. The latter increases the effects of the upstream fibers on the p_CO_2__ distribution of downstream fibers. In general, the first fiber shows the thinnest boundary layer. The boundary layer becomes thicker the further downstream the fiber is positioned. For the relatively homogenous flow distribution observed in this simplified packing, the average of all eight fibers gives a reasonable representation of the single p_CO_2__ profiles of the individual fiber positions (Figure 14). Consequently, in the following graphs, only the average of all eight fiber profiles are shown in order to maintain clarity.

**Figure 15:**
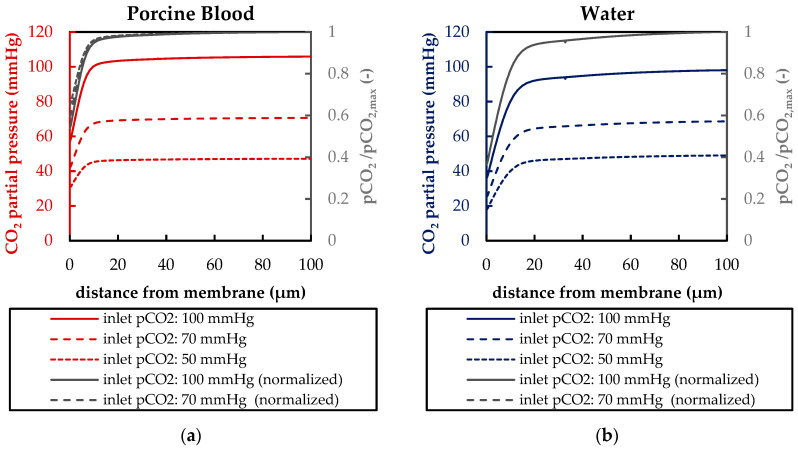
Boundary profiles of CO_2_ partial pressure (pCO_2_) and normalized CO_2_ partial pressure (p_CO_2__^′^) (sample line illustrated in Figure 7a) at different inlet p_CO_2__ and an inlet velocity of 0.02 m/s (approx. 1300 mL/min): (**a**) porcine blood (**b**) water (normalized profiles are overlapping).

Figure 15 compares the average p_CO_2__ boundary layer profiles for the three different inlet p_CO_2__ levels (50, 70, 100 mmHg) at an inlet velocity of 0.02 m/s (approx. 1300 mL/min). With decreasing distance to the hollow fiber membrane, the p_CO_2__ decreases slowly at first, but drops steeply in the last section toward the fiber. This steep drop starts at comparable positions independent from the inlet p_CO_2__. Hence, the p_CO_2__ gradient of this section deviates strongly between the different investigated inlet CO_2_ partial pressures. The gradient of p_CO_2__ equals, on average, 3.5 mmHg/µm for an inlet p_CO_2__ of 100 mmHg and 1.7 mmHg/µm for an inlet p_CO_2__ of 50 mmHg.

Additionally, the normalized p_CO_2__ profiles (p_CO_2_′_(**x**)) are given. They are calculated by dividing the p_CO_2__ at any point in the packing (p_CO_2__(**x**)) by the maximum p_CO_2__ in the bulk flow (p_CO2,max_), which is equal to the p_CO_2__ at the inlet (p_CO_2__,_inlet_—Equation (6).

(8) $$p_{{CO}_{2}\left(X\right)}^{’}=\frac{p_{{CO}_{2}\left(x\right)}}{p_{{CO}_{2},max}}=\frac{p_{{CO}_{2}\left(x\right)}}{p_{{CO}_{2},inlet}}$$

While for water the normalized p_CO_2__ profiles are very similar for all three inlet p_CO_2__ (overlapping of all three dimensionless profiles, Figure 15b), the normalized p_CO_2__ profiles for blood deviate slightly (Figure 15a). However, p_CO_2_′_ is capable of giving a reasonable representation of the p_CO_2__ boundary layers at different inlet CO_2_ partial pressures. 

**Figure 16:**
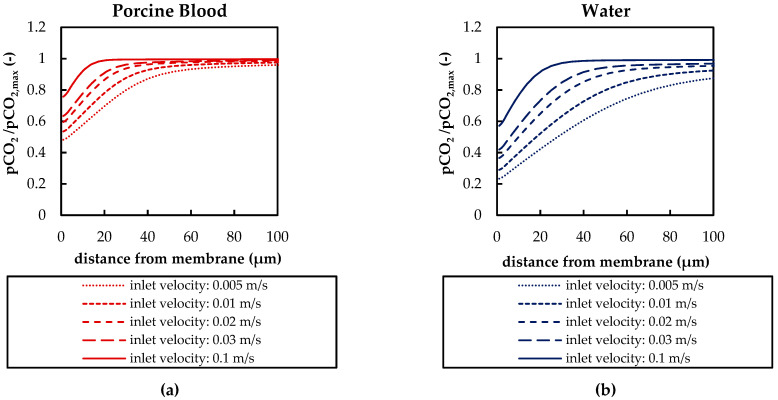
Boundary profiles (sampling line illustrated in Figure 7a) of normalized CO_2_ partial pressure (pCO_2_/pCO_2,max_) for different inlet CO_2_ partial pressures: (**a**) porcine blood, (**b**) water.

Figure 16 compares the dependence of the p_CO_2__ boundary layer profiles on the inlet velocities of the simplified packing for porcine blood and water. Five inlet velocities (0.005, 0.01, 0.02, 0.03, 0.1 m/s) were simulated. With decreasing inlet velocities, the boundary layers become thicker and the p_CO_2__ gradients smaller. The principal shape of the profiles differs depending on whether higher or lower flow velocities are present. At higher inlet velocities (≥0.1 m/s), there is a low decrease in p_CO_2__ with decreasing fiber distance followed by a steep decrease. At lower inlet velocities (≤ 0.005m/s), p_CO_2__ decreases gradually with fiber distance. In general, water shows thicker p_CO_2__ boundary layers than blood and a more pronounced dependence of the p_CO_2__ boundary layer on the inlet velocity.

**Figure 17:**
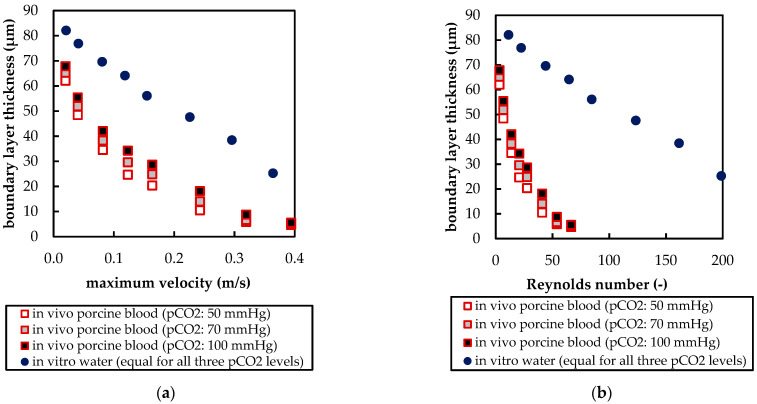
Dependency of boundary layer thickness on: (**a**) maximum velocity between fibers; (**b**) Reynolds number computed with maximum velocity between fibers.

Figure 17 compares the boundary layer thickness (δ) for blood and water at different velocities. The end of the boundary layer was defined at the fiber distance (x) where 99% of bulk flow p_CO_2__ was reached (Equation (7)) [28]. The fiber distance and p_CO_2__ were taken along the sample line, illustrated in Figure 7a. For p_CO_2__, average profiles of all eight fibers were used.

(9) $$\delta =x\left(p_{{CO}_{2}}\left(x\right)=0.99\times p_{{CO}_{2},inlet}\right)$$

In Figure 17a, δ is plotted over the maximum velocity between fibers. The boundary layer thickness of porcine blood is smaller and, in contrast to water, dependent on inlet p_CO_2__. At elevated velocities, this dependency becomes less. In Figure 17b, δ is plotted over the Reynolds number (Re). Re was calculated Equation (8) using the fiber diameter (d_fiber_), maximum velocity between fibers (u_max_), and, due to elevated shear rates > 400 s^−1^ in larger parts of the geometry, Newtonian kinematic viscosity (*ν*) of the respective fluids (porcine blood or water, Figure 2).

(10) $$Re=\frac{u_{max}\times d_{fiber}}{\nu }$$

In general, water shows a higher Re than porcine blood due to its lower viscosity. The boundary layer thickness deviates more strongly when comparing porcine blood and water at the same Re (Figure 17b) than when comparing at the same maximum velocities (Figure 17a). This indicates that at similar velocities, additional mixing, promoted by lower viscosity of water (higher Re), does reduce the difference in the boundary layer thickness between blood and water.

**Figure 18:**
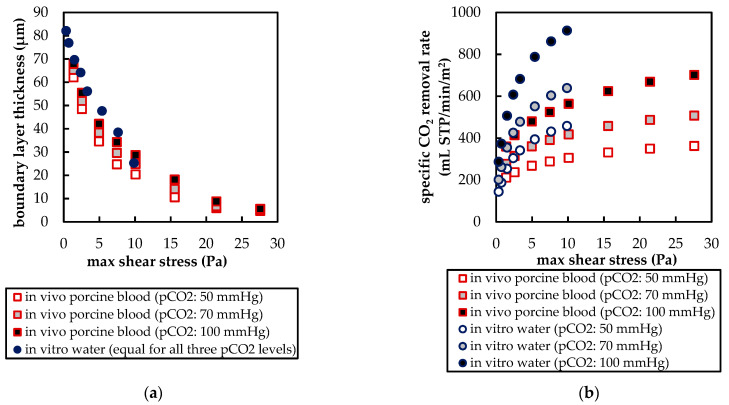
Influence of maximum shear stress on: (**a**) boundary layer thickness; (**b**) specific CO_2_ removal rate.

The boundary layer thickness of porcine blood and water at the same maximum shear stress agree reasonably (Figure 18a), confirming that boundary layer thickness is mainly dependent on shear stress [29]. However, when comparing the CO_2_ removal rates of porcine blood and water at same shear stresses (Figure 18b), the deviation is stronger (min. deviation 20% for max shear stress > 1 Pa) than when compared at the same blood/water flow rates (approx. 10%, Figure 9). Consequently, the boundary layer thickness and CO_2_ removal rate cannot be matched simultaneously.

The differences between the boundary layer mass transfer characteristics of porcine blood and water can be confirmed by establishing dimensionless Sherwood correlations. The mass transfer analogy for crossflow within hollow fiber membrane packings is usually expressed by the dimensionless Sherwood (Sh) Reynold (Re) and Schmidt (Sc) number, as well as the empirical parameters a and b [27]:

(11) $$Sh=a\times Re^{b}\times Sc^{0.33}$$

To account for the diffusion of bicarbonate and physically dissolved CO_2_ in blood, the definitions of Sh and Sc proposed by Federspiel et al. [11] were used. Sh is calculated using the CO_2_ mass transfer coefficient (k_CO_2__), fiber diameter (d_fiber_), CO_2_ solubility in blood (α_CO_2__, Table 4), and facilitated diffusion of CO_2_ in blood (D_f_).

(12) $$Sh=\frac{k_{{CO}_{2}}\times d_{fiber}}{\alpha_{{CO}_{2}}\times D_{f}}\;$$

Sc number is defined by the Newtonian kinematic viscosity of blood (ν_blood_) and effective diffusion of CO_2_ in blood (D_eff_).

(13) $$Sc=\frac{\nu_{blood}}{D_{eff}}$$

The Sh and Sc numbers of water can be calculated analogously. Here, facilitated and effective diffusivity are replaced with the diffusivity of CO_2_ in water (Table 4). The CO_2_ mass transfer coefficient was determined with the numerically predicted average CO_2_ flux (j_CO_2__) and the CO_2_ partial pressure difference between the blood/water bulk flow and gas side (Δp_CO_2__):

(14) $$k_{{CO}_{2}}=\frac{j_{{CO}_{2}}}{\Delta p_{{CO}_{2}}}$$

The Re number was calculated according to Equation (10). The definitions of D_f_ and D_eff_ are given in [11].

**Figure 19:**
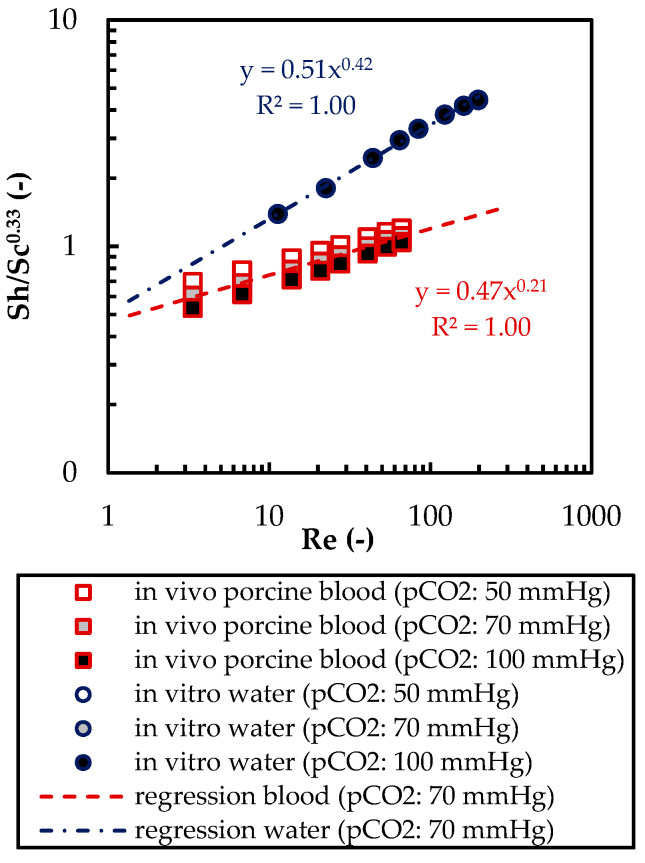
Sherwood correlations for porcine blood and water determined via the CFD results.

Comparison of the Sherwood analogies (Figure 19) shows different correlations for porcine blood and water. While parameter a of porcine blood (0.47) and water (0.51) is comparable, parameter b deviates by a factor of 2 (b porcine blood: 0.21, b water: 0.42). This leads to agreement between the two correlations only for Re numbers of about 1. The varying values of Sh/Sc^0.33^ at different p_CO_2__ for porcine blood are caused by the non-linear dependency of the CO_2_ partial pressure and CO_2_ concentration (c_CO_2__) and the definition of k_CO_2__, which uses p_CO_2__ instead of c_CO_2__ as the driving force (Equation (14)). Based on the available data, CO_2_ mass transfer within the boundary layer of porcine blood and water does not exhibit similar characteristics.

#### 3.2.3. Influences of Fluid Properties on Boundary Layer Thickness

In further CFD studies, the dependency of the boundary layer thickness on important fluid and material parameters was examined. Therefore, the original CFD model for water was adapted by singularly modifying the CO_2_ diffusion coefficient (CO_2_ diff. coeff.), CO_2_ permeance, CO_2_ solubility and the viscosity model to the properties of blood. 

**Figure 20:**
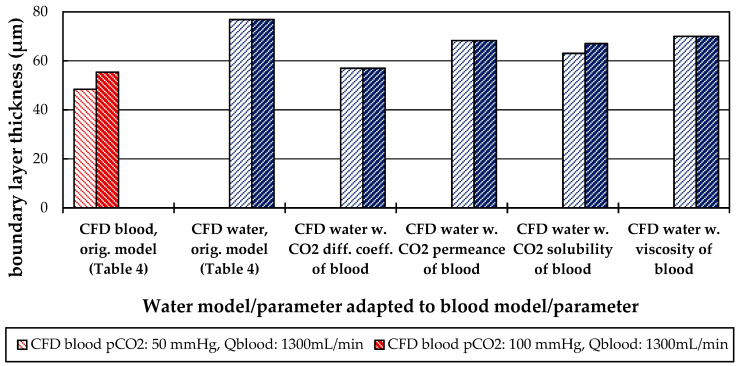
Influence of CO_2_ diffusion, CO_2_ permeance, CO_2_ solubility, and viscosity model on the boundary layer thickness.

The boundary layer thicknesses, determined with these modified CFD models, are compared in Figure 20 for a flow rate of 1300 mL/min and CO_2_ partial pressures of 50 and 100 mmHg. As reference, the boundary layer thicknesses determined with the original CFD models are entered. A summary of the model and parameter values can be found in Table 4.

Modification of the water CFD model by changing individual fluid and material parameters to those of blood resulted in a reduction in boundary layer thickness in all of the studied cases (Figure 20). The strongest dependency of boundary layer thickness was detected for adaption of the CO_2_ diffusion coefficient from a value of 2.38 × 10^−9^ m^2^/s (CO_2_ in water) to 6.96 × 10^−10^ m^2^/s (CO_2_ in blood). By this adaption, the boundary layer thickness reduced to 57 µm, which is comparable to porcine blood with a boundary layer thickness of 55 µm at 100 mmHg inlet p_CO_2__. The reduction in boundary layer thickness induced by CO_2_ permeance, CO_2_ solubility model and viscosity is less pronounced. The boundary layer thickness determined with these simulations is similar and approximately 68 µm. 

Of all model adaptions, only the CO_2_ solubility model of blood introduces a dependency of the boundary layer thickness on the inlet p_CO_2__. This is probably due to the binding of CO_2_ in multiple components (physically dissolved, bicarbonate and carbaminohemoglobin) and the resulting nonlinear dependency of the CO_2_ partial pressure from the CO_2_ concentration (Figure 1a).

#### 3.2.4. Influences of Fluid Properties on CO_2_ Removal Rate

Additional CFD studies were conducted to examine the influence of the CO_2_ diffusion coefficient (CO_2_ diff. coeff.), CO_2_ permeance, CO_2_ solubility and the viscosity model on the specific CO_2_ removal rate. To do so, the original CFD model for water was adapted by singularly modifying the fluid and material parameters to those of blood. 

**Figure 21:**
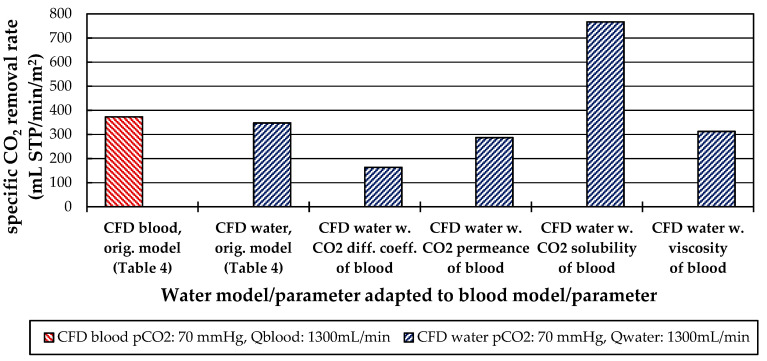
Influence of CO_2_ diffusion, CO_2_ permeance CO_2_ solubility, and viscosity model on the CO_2_ removal rate.

Figure 21 compares the specific CO_2_ removal rate at a flow rate of 1300 mL/min and a CO_2_ partial pressure of 70 mmHg, determined with the modified and original CFD models for blood and water. As can be seen in Figure 21, the CO_2_ removal rates of blood and water are comparable despite the different fluid and material properties (Table 4). This is due to their opposing effects on the CO_2_ removal rate. While the higher CO_2_ solubility of blood allows higher CO_2_ removal rates, the slower CO_2_ diffusion in blood, the lower permeance of membranes contacted with blood and the higher blood viscosity lower the CO_2_ removal rate. The strongest effect on the CO_2_ removal rate is caused by the difference in CO_2_ solubility between blood and water (121% increase), followed by the difference in CO_2_ diffusion rate (53% decrease) and the difference in CO_2_ permeance (18% decrease). Based on the CFD results, the rheologic difference between blood and water has the smallest effect on the CO_2_ removal rate (10% decrease).

## 4. Conclusions

The CO_2_ removal rate of a prototype oxygenator was measured in vivo using porcine blood and in vitro using water. In general, the CO_2_ removal rates of porcine blood and water are comparable despite differing fluid properties (CO_2_ diffusion, CO_2_ solubility and viscosity). The deviation of the CO_2_ removal rate determined with porcine blood from that determined with water amounts to approximately 10%. This deviation agrees well with the data found in recent literature [20,21,22]. Based on the results of the in vivo and in vitro tests, the CFD CO_2_ transport simulations were validated. 

Besides differences in the fluid properties of blood and water, our experimental and simulation data indicate that additionally higher CO_2_ permeances are available during tests with water, probably due to the absence of blood residues on the membrane surface. The influences of the differing fluid properties (CO_2_ diffusion, CO_2_ solubility and viscosity) as well as membrane material properties (CO_2_ permeance) on CO_2_ removal were quantified, utilizing the developed CFD models. The difference in CO_2_ solubility between blood and water has the strongest effect on the CO_2_ removal rate, followed by the difference in the CO_2_ diffusion rate, difference in CO_2_ permeance and difference in viscosity. 

The CFD simulations also allow to resolve and study the main CO_2_ transport resistance—the diffusional boundary layer attached to the membrane surface. The simulations show that the p_CO_2__ boundary layer in water is, in general, thicker than that in blood. The CFD results indicate that the thicker boundary layer in water can be mainly attributed to the higher diffusion coefficient of CO_2_ in water (2.38 × 10^−9^ m^2^/s) than in blood (6.96 × 10^−10^ m^2^/s). Furthermore, the CFD model suggests that the p_CO_2__ boundary layer thickness in blood is dependent on bulk p_CO_2__. This is probably due to the binding of CO_2_ in multiple components (physically dissolved, bicarbonate and carbaminohemoglobin) and the resulting nonlinear dependency of the CO_2_ partial pressure from the CO_2_ concentration (CO_2_ solubility). Additionally, CFD simulations indicate that the p_CO_2__ boundary layer thicknesses of porcine blood and water are in good agreement when compared at same shear stresses. However, the CO_2_ removal rates of porcine blood and water deviate stronger at the same shear stresses (>20%) than at the same blood/water flow rates (approx. 10%). Consequently, the boundary layer thickness and CO_2_ removal rate cannot be matched simultaneously. Differences in the CO_2_ mass transfer characteristics of the boundary layer can be confirmed when comparing Sherwood correlations established for porcine blood and water.

To conclude, the boundary layers of blood and water behave, in general, differently. Studies of blood oxygenators aiming to investigate the boundary layer or measures for boundary layer reduction should, therefore, rely on blood tests. However, the determination of the total CO_2_ removal rate of an oxygenator using water as a blood substitute should be possible with reasonable accuracy. Deviation of the CO_2_ removal rate determined with water to the CO_2_ removal rate determined with blood should lie within 10%. This level of accuracy could potentially differ for flow conditions other than those studied here (crossflow, Re within the packing approx. 20). Nevertheless, the results of this work provide a basis for future optimization of the CO_2_ removal performance of oxygenators, using in vitro tests with water.

## Figures and Tables

**Table 1:** Boundary conditions of the flow simulations.

Boundary	Velocity	Pressure
Inlet	uniform constant value	zero gradient ^1^
Outlet	zero gradient ^1^	uniform constant value
Membrane	no-slip	zero gradient ^1^
Walls	no-slip	zero gradient^1^

^1^ Equal to Neumann boundary condition.

**Table 2:** Inlet velocities of reduced geometry determined with up-scaling method [8].

Total Flow Rate (mL/min)	Inlet Velocity Blood (m/s) ^1^	Inlet Velocity Water (m/s) ^1^
1000	0.015	0.0148
1300	0.024	0.0192
1600	0.031	0.0237

^1^ Inlet velocity of reduced geometry representative for the total flow rate in prototype oxygenator.

**Table 3:** Boundary conditions of the CO_2_ transport simulations.

Boundary	Velocity	Pressure	CO_2_
Inlet	uniform constant value	zero gradient ^1^	uniform constant value
Outlet	zero gradient ^1^	uniform constant value	zero gradient ^1^
Membrane	uniform constant value	zero gradient ^1^	zero gradient ^1,2^
Sides	symmetry	symmetry	symmetry

^1^ Equal to Neumann boundary condition. ^2^ Transmembrane transport applied as source term in the cell center.

**Table 4:** Comparison of parameter values for blood and water used in CO_2_ transport simulations.

Symbol	Description	Value Blood [8]	Value Water	Unit
α_CO_2__	CO_2_ solubility	8.77 × 10^−3 (1)^	8.27 × 10^−4^	mL CO_2_/mL/mmHg
D_CO_2__	CO_2_ diffusion coefficient	5.05 × 10^−10 (2)^	2.38 × 10^−9^	m^2^/s
µ	dynamic viscosity	2.38 × 10^−3 (3)^	6.91 × 10^−4^	Pa s
ρ	density at 37 °C	1.05 × 10^3^	9.93 × 10^2^	kg/m^3^
P_CO_2__	CO_2_ permeance of used fibers	157	275	GPU

^1^ At 70 mmHg. ^2^ Diffusion of total CO_2_ (bicarbonate + physically dissolved CO_2_). ^3^ At shear rates > 400 1/s.

**Table 5:** Deviation of the in vitro CO_2_ removal with water to the in vivo CO_2_ removal with blood (ε).

Flow Rate ^1^	Deviation (ε) of In Vitro CO_2_ Removal (Water) to In Vivo CO_2_ Removal (Blood)		
(mL/min)	pCO_2_: 50 mmHg	pCO_2_: 70 mmHg	pCO_2_: 100 mmHg
1000	21.8%	8.6%	9.7%
1300	9.9%	10.6%	6.7%
1600	2.1%	7.9%	9.6%

^1^ Flow rate of blood or water on prototype oxygenator shell side.

## Data Availability

The data that support the findings of this study are available from the corresponding author upon reasonable request.

## Nomenclature

**Acronyms**	
CFD	Computational fluid dynamics
CO_2_	Carbon dioxide
H^+^	Hydronium ion
H_2_CO_3_	Carbonic acid
HCO_3_^−^	Bicarbonate
N_2_	Nitrogen
O_2_	Oxygen
**Latin Symbols**	
A	Membrane area
a	Empirical parameter of Sherwood correlation (multiplier)
b	Empirical parameter of Sherwood correlation (exponent)
D_CO_2__	CO_2_ diffusion coefficient
D_eff_	Coefficient of effective diffusion of CO_2_ in blood
D_f_	Coefficient of facilitated diffusion of CO_2_ in blood
d_fiber_	Outer hollow fiber membrane diameter
J_CO_2__	Transmembrane CO_2_ transport
j_CO_2__	Transmembrane CO_2_ flux
J_CO_2_,blood_	Transmembrane CO_2_ transport in in vivo porcine blood tests
J_CO_2_,water_	Transmembrane CO_2_ transport in in vitro water tests
k_CO_2__	CO_2_ mass transfer coefficient
L	Characteristic length of the Reynolds number
P	Membrane permeance
p	Pressure field
P_CO_2__	CO_2_ membrane permeance
p_CO_2__	CO_2_ partial pressure
p_CO_2_,inlet_	Inlet CO_2_ partial pressure
p_CO_2_,max_	Maximum CO_2_ partial pressure
p_CO_2_,water_	CO_2_ partial pressure in water
p_CO_2__‘	Normalized CO_2_ partial pressure
Re	Reynolds number
Re_inlet_	Reynolds number at the inlet pipe of the prototype oxygenator
Re_packing_	Reynolds number in the hollow fiber packing of the prototype oxygenator
Sc	Schmidt number
Sh	Sherwood number
**U**	Velocity field
u	Characteristic velocity of Reynolds number
u_max_	Maximum velocity between two fibers
**x**	Arbitrary point on shell side of prototype oxygenator
**x**_inlet_	Arbitrary point on shell side at inlet of prototype oxygenator
**Greek Symbols**	
α_CO_2__	CO_2_ solubility
Δp_CO_2__	CO_2_ partial pressure difference between blood bulk flow and gas side
δ	CO_2_ boundary layer thickness
ε	Relative deviation of in vivo porcine blood to in vitro water CO_2_ removal rate
µ	Dynamic viscosity
ν	Kinematic viscosity
ρ	Density
